# Integration of resources in social and healthcare services for ensuring the continuity of care to frail individuals aged 65 or over: an Italian experience

**DOI:** 10.3389/fpubh.2025.1562564

**Published:** 2025-05-21

**Authors:** Martina Giusti, Santa De Remigis, Stefano Greco, Valerio Filippo Profeta, Guglielmo Bonaccorsi, Patrizio Zanobini, Marco Del Riccio, Chiara Lorini, Maurizio Di Giosia, Niccolò Persiani

**Affiliations:** ^1^Department of Experimental and Clinical Medicine, University of Florence, Florence, Italy; ^2^Centro Studi SAPIS Foundation, Italian National Federation of Orders of Radiographers and Technical, Rehabilitation, and Prevention Health Professions Research Centre, Rome, Italy; ^3^Local Health Authority of Teramo, Teramo, Italy; ^4^Department of Health Sciences, University of Florence, Florence, Italy

**Keywords:** frail individuals aged 65 or over, continuity of care, integrated social-health care, organizational model, collaborative network

## Abstract

**Introduction:**

Globally the ongoing reforms of the national healthcare systems are carrying out social and health services closer to users, increasingly reaching them at home. This approach addresses the challenges posed by population aging, chronic diseases, and social inequalities. Effective solutions require combining experience, expertise, and resources within a unified organizational model for the social and health care services provision. This is particularly crucial for providing integrated care to frail individuals aged 65 or over. This article aims to explore service innovations for frail population aged 65 years or older, focusing on the integrated management of economic and professional resources in health and social services to ensure continuity of care.

**Method:**

The Agency of Integration for Continuity between Hospital and Territory (AgICOT), a service financed by the Italian Ministry of Health designed for frail individuals aged 65 or over in Local Health Authority of Teramo (Abruzzo, Italy) was selected as case study.

**Results:**

AgICOT utilized health plans and health budgets to integrate social and health goals and resources into a single social and health care pathway, avoiding duplication of interventions, saving resources, and ensuring economic sustainability of social and healthcare systems.

**Discussion:**

AgICOT has demonstrated its effectiveness as organizational response to the complex needs of frail individuals aged 65 or over, ensuring high-quality social and healthcare services in a sustainable manner.

**Final considerations:**

The co-responsibility of various stakeholders has been the key to the success of this project, creating the conditions for transitioning from hospital-based to person-based social and healthcare in line with international recommendations on integrated care and continuity of care.

## 1 Introduction

The improvement of life expectancy, driven by enhanced social and economic conditions, has led to unprecedented population aging. This demographic shift presents significant challenges in providing adequate care for individuals aged 65 or over who often have complex social and health needs (1). International organizations and national governments are working toward future healthier population by addressing determinants of health—non-medical factors influencing health outcomes—(2). However, social and healthcare systems continue to struggle with managing older adults taking care who require resources-intensive responses to adequately meet their needs.

This has created sustainability challenge for both social and health services (3). Addressing these challenges requires comprehensive strategies that consider both social and health determinants while integrating services to meet evolving demands effectively (4).

For example, Italy ranks as the second oldest country in the world, with 24.3% of its population aged 65 or over (5). While this reflects the Italian Welfare State's ability to ensure longer life expectancy than the global average, many older adults experience chronic illnesses during the final years. Data from 2022 to 2023 evidence that 59% of individual aged 65 or over have been diagnosed with one or more chronic conditions such as cardiovascular diseases (28%), respiratory diseases (17%), diabetes (20%), and cancers (14%). Multiple chronic diseases affected 1 in 4 individuals aged 65 or over, with prevalence increasing with age (17% among those aged 65–74 vs. 38% aged 85 or over) and decreasing with socioeconomic status (41% among those facing financial difficulties vs. 19% among those without; 31% lower education levels vs. 19% higher education levels). No significant gender differences were observed (6). So, the demand for social and health care from individuals aged 65 or over who are sick, multi-pathological, and vulnerable individuals—often struggling to access integrated health and social services—has been increasing (7).

Globally, government reforms have aimed not only at reorganizing hospital networks but also at enforcing continuity of care between hospitals, primary health care centers, and social services due to the growing need for integration from clinical to organizational perspective, at the same time ensuring economic sustainability of the social and health care systems (8, 9).

The transitional care (TC) approach encompasses actions designed to ensure coordination and continuity in social-healthcare delivery. This approach includes logistical arrangements, patient/family education, and seamless coordination among professionals involved in the patient transitions—all contributing to a patient-centers social and health care model (10, 11). Studies highlight that integrating social-health services through co-designing personalized care pathways improves resource allocation while enhancing assisted individuals' outcomes and satisfaction (12).

Despite clear recommendations from scientific community regarding integrated care based on PHC and TC principles to address aging population, chronic diseases, and inequalities effectively, these recommendations have only been partially implemented due to the lack of consolidated procedures and protocols to provide integrated social and health services at national level (13).

Always referring to Italian context, over the past decade, Primary Health Care have been progressively strengthened (14) to promote integrated care (15) addressing population aging, chronic diseases, social inequalities both at national and regional levels (16–18) in alignment with international strategies (2, 3). Currently, it is ongoing the reform of Italian national healthcare system proposed in Mission 6 “Health” within the National Plan for Recovery and resilience (19). The main goal of this reform in strengthening community-based health care services, extending the types of local social-health facilities within a Local Health Authority (not only social-health districts and family centers but also community houses—CdC, community hospitals—OdC, and community-based operative centers—COT) and overcoming the hospital-centered approach in social and healthcare.

The COVID-19 pandemic exacerbated this issue by disrupting continuity-of-care services almost entirely and dramatically impacting life expectancy (20). In 2020 alone, life expectancy fell by an average of 0.6 years across countries belonging to the Organization for Economic Cooperation and Development (OECD), dropped by as much as 1.2 years in Italy (21, 22).

The awareness of having disregarded international recommendations—mainly in relation to the absence of national pandemic plans- for health protection has prompted a revaluation of alternative organizational and integrative approaches for the provision of social and healthcare services, especially in Europe. This revaluation has focused on the development of public-private network-based social and healthcare service provision (23–25). A network-based welfare system involves mixed management and shared responsibility among public institutions, private entities, patients, families, and communities (26).

This organizational evolution has been favored by the implementation of innovative tools. Among these, the Personal Health Budget (PHB) stands out as one of the most significant and widely adopted tool in Europe (27). The PHB is an accounting mechanism that determinates the quantity and type of resources—economic, professional, and/or human—that stakeholders allocate collectively to reach specific social and health needs. It enables individuals to access services that best align with their personalized Health Plan (28). The HP is a mandatory document that outlines the social and healthcare pathway for each individual. It is developed through a joint multidimensional assessment by social and healthcare services and agreed upon with the beneficiary (29). The positive impact of using a PHB is maximized when supported by a care planner or broker. This professional assists patients throughout their HP journey ensuring full adherence to the plan. This profile helps patient navigate bureaucratic obstacles, provides additional information, when needed (30), and performs second-level checks to monitor any misuse or underutilization of allocated resources in relation to the HP (30). This process ensures that unused resources can be reallocated efficiently, contributing to resources saving. Both HP and PHB place the preference of assisted individual at the center of care, moving away from organizational or managerial constraints (31). These tools allow individuals of all ages, with diverse social and health challenges, to maintain autonomy in decision-making and experiment with self-management (32).

Internationally, the most structured and exemplary application of HPs and PHBs are applied found in the United Kingdom (33). In this context, PHBs do not involve cost-sharing by welfare state for providing social-health services remaining the same concept of HPs. Instead, an Integrated Health Budget (34)—co-funded by English NHS and welfare state, is available only for individuals in a highly vulnerable state, both socially and health-wise; the IHB aims, in fact, to ensure equitable care for all patients regardless of their socio-economic status (35).

While tools like HPs and PHBs are instrumental in advancing integrated care, a pressing challenge remains ensuring their interoperability within existing information systems used in health and social services to reach required standards-based roadmaps that enables seamless interconnection between these sectors (36). In 2023, the WHO published the document “Global strategy on digital health 2020–2025” (37, 38), which inspired ISO 13940:2015 on “Health informatics—System of concepts to support continuity of care” (12, 39). This framework highlights interoperability as a cornerstone for efficient integration between health and social care services.

In Italy, the law 77/2022 entitled “Regulation on the definition and standards for the development of territorial assistance in the national health service” presents the organizational model to implement of Mission 6 “Health” within PNRR but the relational and organizational infrastructure between different social and healthcare facilities, included assisted person's home, is still mainly theoretical.

Despite numerous studies analyzing the benefits of integrating social and health services (40, 41), a knowledge gap remains regarding services innovations from organizational and managerial perspectives—particularly concerning frail individuals aged 65 or over (39, 42, 43).

This study aims to explore public service innovations in continuity of care of frail individuals aged 65 or over, focusing on the integrated management of economic and professional resources in social and health care services.

## 2 AgICOT as community-based study

### 2.1 Empirical approach

The case study methodology was deemed the most suitable approach for achieving the objectives of this community-based study (44, 45). This method enables an in-depth understanding of phenomena within their intervention context (46), particularly when the object of analysis is complex—such as the study and the evaluation of the integrated management of economic and professional resources in the social and health care of frail individuals aged 65 or over.

Among several national initiatives aimed at integrating social and healthcare services for frail individuals aged 65 or over, the Italian governmental project for the establishment of Agency of Integration for Continuity between Hospital and Territory (AgICOT) in Abruzzo Region was selected as significant case study. AgICOT has been recognized as a national best practice by the National Agency for Regional Health Services (AGENAS).

A key strength of this methodology lies in its ability to examine phenomena from a practical perspective, addressing one of the most discussed issues in literature: the gap between theory and practice (47). The AgICOT project represents an innovative public service capable of integrating economic and professional resources for social and health care to frail individuals aged 65 or over in an effective collaborative network, where all stakeholders have been involved. This was achieved by fostering shared knowledge, language, and practices among social and healthcare professionals thought joint training on topics of mutual interest.

Ultimately, the case study approach facilitates the generalization of findings, broadening their applicability to comparable scenarios (48). The AgICOT governmental project was initially tested as a pilot study within the Local Health Authority of Teramo (ASL Teramo). After being implemented, evaluated, and optimized at regional level in Abruzzo, AgICOT could serve as positive model of continuity of care for national application.

#### 2.1.1 Data collection

Following an investigation of the regional (Abruzzo Region) and local (ASL Teramo) contexts to provide background information, operational data regarding the assessment and implementation phases of the AgICOT project were gathered thorough semi-structured interviews with involved managerial and professional profiles staff. The following individuals were interviewed to represent the managerial perspective:

- The general manager of ASL Teramo (Dr. M.D.G., administrative), who consistently supported the AgICOT project within the Local Health Authority he manages, providing ongoing endorsement.
- The scientific coordinator of the AgICOT project for ASL Teramo (Dr. V.F.P., doctor), who oversaw the reorganization of ASL Teramo services to ensure the effective implementation of the AgICOT project.

The professional perspective was represented, indeed, by:

- The coordinator of the ASL Teramo working group (Dr. S.D.R., doctor), who managed the administrative, training, and organizational activities required to establish the AgICOT service.
- The AgICOT service referent (Dr. S.G., doctor), who managed AgICOT during its operational period.

These interviews explored:

- Strategic objectives related to continuity of care within the area of operation.
- Establishment of AgICOT.
- AgICOT activities.
- AgICOT impact.

A coding method was employed to analyse and synthesize the qualitative data obtained from semi-structured interviews (49, 50). All participants were required to sign a privacy policy document to consent to the management of their personal data (51, 52). Aggregate data on the volume and types of activities carried out by AgICOT were shared by the management of the ASL Teramo, extracted from the digital platform system used in AgICOT service call “2CARE” by Kell company.

Additionally, data were acquired through the analysis of secondary sources, including regional normative and administrative documents (e.g., company acts, deliberations) and position papers related to strengthening continuity of care in Abruzzo, especially in the province of Teramo (53, 54).

Data collected from both primary and secondary sources facilitated an in-depth study of the tools adopted for the integrated management of economic and professional resources in the social and healthcare of frail individuals aged 65 or over within the AgICOT project.

### 2.2 The case study AgiCOT

In Italy, the organizational innovation of the Agency of Integration for Continuity between Hospital and Territory, known as AgICOT, was the object of study.

This Agency was established in response to the Italian Ministry of Health's notice entitled “Guidelines for the implementation of regional projects on the experimentation of proximity structures” (Law Decree 34/2020, later Law 77/2020).

AgICOT was financed by Italian Ministry of Health to be developed by the Abruzzo Region with scientific support from the University of Florence and was tested over three years (2021–2024) in Local Health Authority ofTeramo (ASL Teramo)^1^.

The AgICOT project was launched in ASL Teramo because this Local Health Authority could immediately fully satisfy the prerequisites specified by the governmental notice in term of experienced services for continuity of care.

AgICOT's scope was to introduce an innovative organizational model of functional aggregation to coordinate the activities of social and healthcare services as a unified network to assist its target population: frail individuals aged 65 or over with a Brass Scale ≥ 10, requiring both social and health interventions, especially during protected hospital discharge.

The integration of responses and resources for the social and healthcare of the frail individuals aged 65 or over, ensuring the continuity of taking charge and monitoring and follow-up over time, was mandatory. To achieve this, AgICOT was equipped with two operational tools to ensure the best possible care of the assisted individuals:

- Health plan (HP): a document that collects clinical, social, and psychological assessments of the individual receiving care. In practice, the HP evaluates functional needs, supports discharge planning, therapeutic reconciliation, and care coordination. From a multidisciplinary perspective, the document gathers clinical, care-related, social, rehabilitative, psychological, and linguistic-communicative evaluations regarding the assisted individual to formulate a comprehensive care project.
- Health budget (HB): a tool for the quantitative and qualitative definition of the economic, professional, human, institutional, and personal resources necessary for socio and healthcare. The HB summarizes and integrates all resources made available by all stakeholders involved (local health authorities, municipalities, as well as the third sector and families/patients).

A dedicated digital platform was implemented to manage social and health information flows, enabling real-time adjustment to the HP and the HB in response to changes in the health or social circumstances of assisted frail individual (Annex 2).

Focusing on the AgICOT's organizational model, particular attention may be given to the core team and the role of case manager. The core team was a multidisciplinary group within AgICOT responsible for conducting multidimensional assessments of the target population's needs and developing HP. This included providing clear quantitative and temporal indications regarding appropriate social and health services and the optimal settings for their delivery. This assessment then informed the definition of the HB. The core team comprised the following professionals:

- Physicians. They coordinate the AgICOT core team, assessed the appropriateness of the referrals, supported the integration of AgICOT with hospital and community-based social and health services, and defined, optimized, activated, and monitored the most suitable HP/HB from available care pathways.
- Social workers. They were responsible for connecting and integrating activities between health services and social services to configure service provision for frail individuals aged 65 or over 65 with a focus on advocacy within civil and legal contexts.
- Coordinator of health professionals. This individual ensured continuity of care across various settings.

When necessary, the core team consulted specialist professionals to determine appropriate responses for assisted individuals.

The case manager, selected from physicians, health professionals, and social workers based on the predominant needs of the assisted individual, was responsible for sharing the HP and HB with the assisted individuals, their caregiver(s) or legal representative(s). The case manager served as primary point of contact within the integrated social-health care system. This role coordinated and guided all the stakeholders -not only the assisted person but also colleagues—throughout the social and healthcare pathway, overseeing adherence to both HP and HB and reporting any changes to the core team over time to facilitate necessary corrections or revisions.

In summary, AgICOT was a service dedicated to providing dignified and appropriate support to its target population until death, transfer to a residence/domicile outside the ASL Teramo service area, or a request for discharge. It integrated social and health responses to avoid inefficiencies and ineffectiveness for assisted individuals and their families favoring a collaborative network among all stakeholders.

## 3 The key factors of AgICOT

### 3.1 AgiCOT activity

From December 2022 to August 2024, AgICOT served 317 frail individuals aged 65 years or older, ensuring integrated continuity of cares across various social and healthcare settings. Of these, 161 were female (51%) and 156 males (49%). A total of 192 (61%) remained active in the program until the AgICOT's closure on August 31^st^, 2024. The remaining 125 individuals (39%) became inactive exiting from the program over time for the different following reasons: i. death (78 individuals, 62%); ii.withdrawal (26 individuals, 21%); iii. non-adherence after initial reporting (11 individuals, 9%); iv. lack of response to contact attempts (6 individuals, 5%); or v. other various reasons (4 individuals, 3%; Table 1).

**Table 1:** AgICOT sample.

**Assisted people**	**Female**	**Male**	**TOTAL**
ACTIVE	96	96	192
INACTIVE	65	60	125
Death	34	44	78
Withdrawal	17	9	26
Non-adherence after initial reporting	5	6	11
Lack of response to contact attempts	2	4	6
Other reasons	2	2	4
TOTAL	161	156	317

ACTIVE, Frail individuals aged 65 years or older accompanied until the AgICOT closing; INACTIVE, Frail individuals aged 65 years or older that left the services before AgICOT closing.

**Figure 1:**
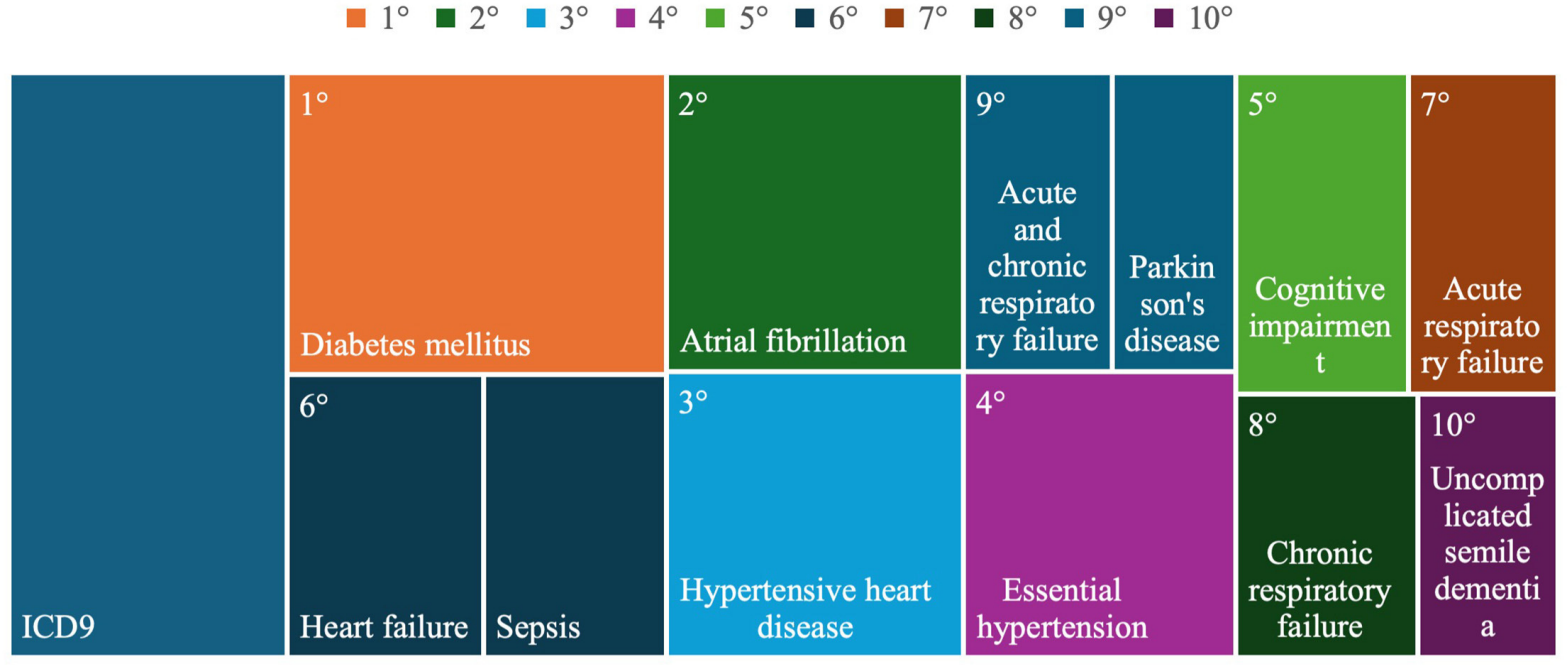
The 10 most frequent pathologies of persons assisted by AgICOT.

The individuals served by AgICOT presented with various co-morbid conditions, with 184 different pathologies identified. The most frequently observed pathologies among AgICOT's target population were diabetes mellitus (44 cases), followed by cardiovascular diseases (atrial fibrillation–31 cases, hypertensive heart diseases–24 cases, and essential hypertension–23 cases), and cognitive impairment (21 cases; Figure 1, Table 2).

**Table 2:** Main pathologies of persons assisted by AgICOT.

**ICD9**	**No**.
Diabetes mellitus	44
Atrial fibrillation	31
Hypertensive heart disease	24
Essential hypertension	23
Cognitive impairment	21
Heart failure	15
Sepsis	15
Acute respiratory failure	14
Chronic respiratory failure	13
Acute and chronic respiratory failure	13
Parkinson's disease	12
Uncomplicated semile dementia	10
Alzheimer's disease	10

AgICOT coordinated and integrated different levels of care, accompanying the assisted individuals in accordance with their HP and supporting them in selecting social and health services to define the HB. This provided appropriate care through timely and periodic monitoring, thereby minimizing resource waste. Operationally, this involved establishing a communicative and collaborative network among all stakeholder to facilitate the provision of the right service at the right time for the assisted individual, who often presented with complex social-health conditions. So, AgICOT's activity primarily focused on integrating and managing social and health care processes, maintaining contact with different actors rather than providing direct frontline care. Case managers monitored adherence both to HP and HB, ensuring the quality of care and timeliness of care provided by social and services providers.

The volume of activity included over 10.000 phone calls and ~330 emails to other services, mainly for booking appointments and/or requesting medical devices. The core team collected, prepared, shared and validated more than 3,800 documents, which were then uploaded to the digital platform (Table 3).

**Table 3:** AgICOT volume of activity.

**Activities**	**No**.
Phone calls	10573
Emails to book visits and/or examination or to request devices	173
Email to communicate with other social and healthcare services	164
Managed documents updated in platform	3819
- Reporting forms	229
- Monitoring forms	2761
- Health Plan/Health Budget (with duplications due to updates)	499
- Hospital discharge forms	185
- Protected hospital discharge forms	53
- Reports of visits/examinations	81
- Other documents	11

**Figure 2:**
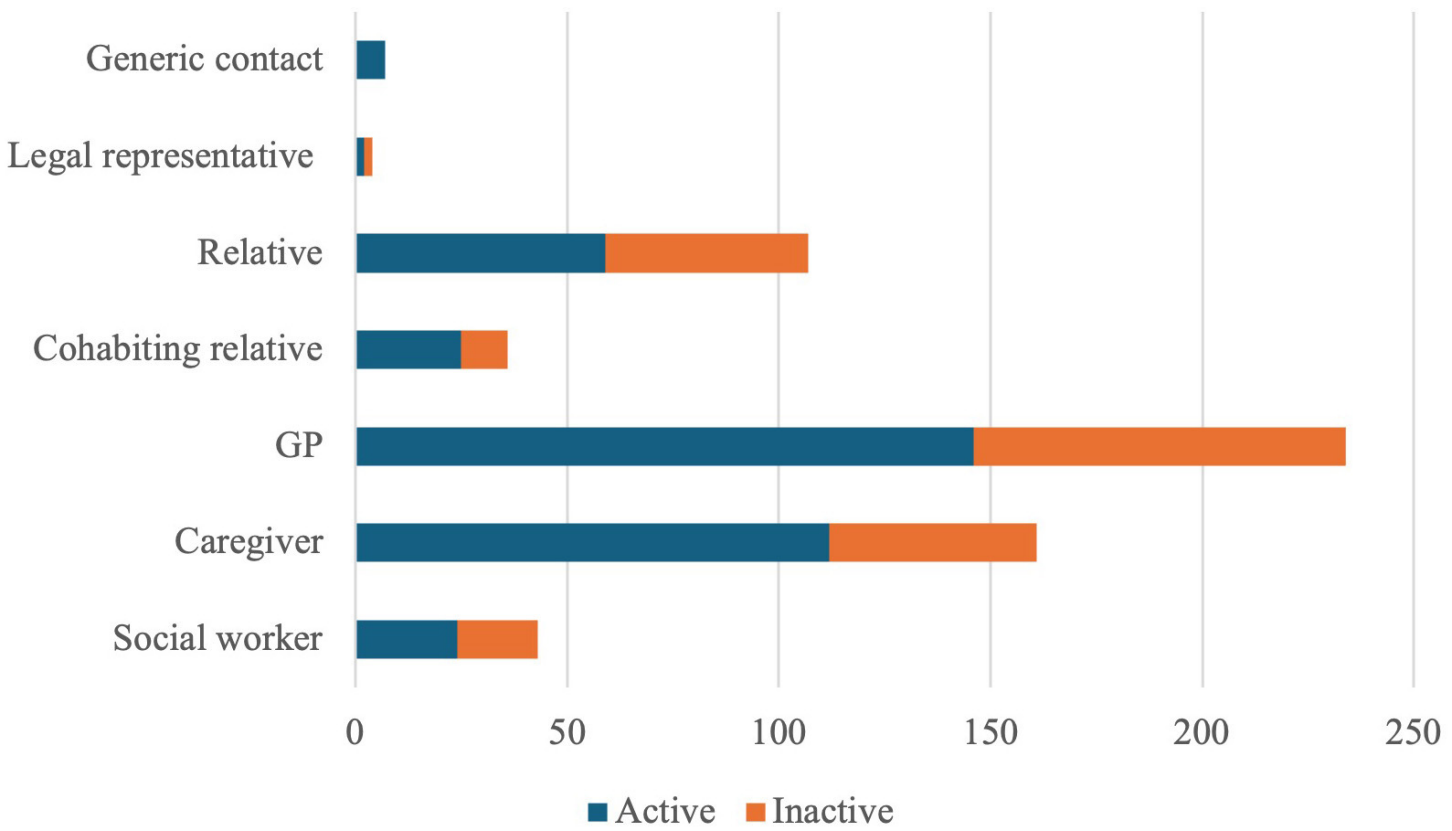
Communication with AgICOT stakeholders.

Regarding external communication, case manager had daily communications with various stakeholder (Figure 2). A strong relationship was especially established with general practitioners and with caregivers of assisted individuals.

### 3.2 AgICOT impact within the ASL teramo

The interviews revealed that the implementation of the AgICOT project, while adhering to all phases outlined in the Italian Ministry of Health's notice, was strategically leveraged by ASL Teramo to accelerate the implementation of Mission 6 “Health” of PNRR (19, 38), and, subsequently, Minister Decree 77/2022 (55) that outlines the Italian government's plan to reform the national healthcare systems by the definition and standards for the development of territorial assistance based on PHC and TC approaches.

**Figure 3:**
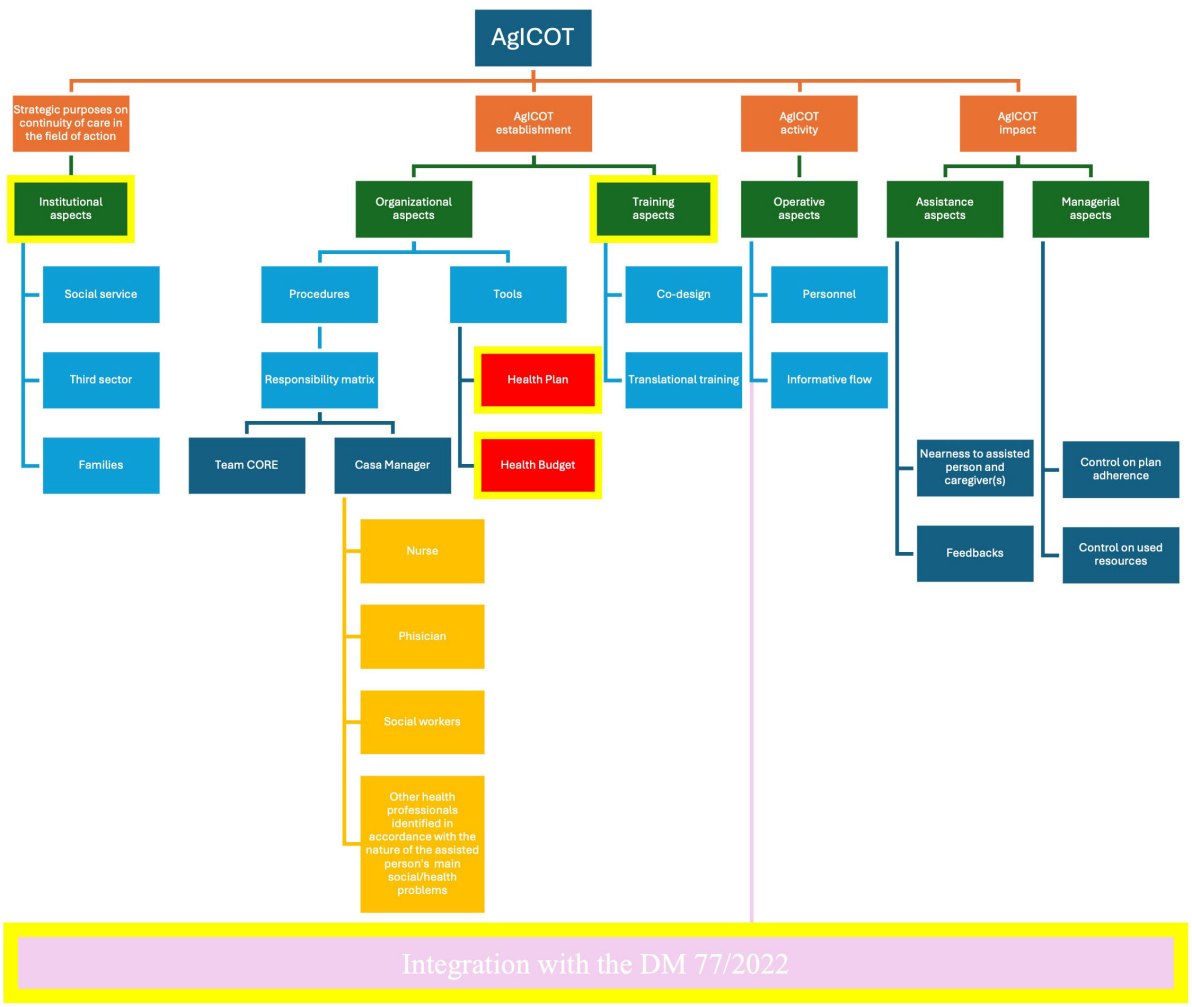
Coding tree by interviews.

All interviewees emphasized how the AgICOT project promoted and expedited the reorganization process of social and healthcare services, yielding significant benefits (Figure 3).

In Annex 3 has been the scheme of semi-structured interview.

The first benefit was the increased time and resources available to manage the transition, enabling substantial investments in strengthening institutional relationships with local partners, such as municipalities and third-sector organizations involved in integrated social and healthcare services.

As part of the AgICOT project, meetings were conducted with:

- Local social districts: resulting from the inclusion of AgICOT in the “Area plans”—a tool to formalize the co-creation and the co-design of integrated social and healthcare services- signed by the ASL Teramo with each one of local social districts in its catchment area.
- Union of municipalities within the catchment area of the ASL Teramo: providing strong endorsement for the AgICOT project by promoting maximum collaboration with municipal social workers. Their work that complemented that of healthcare service colleagues, is essential for the integrated care of assisted individuals.
- Third-sector entities: some of these entities signing agreement to participate actively in the provision of supportive services to socio and healthcare facilities on users accompaniment and orientation, especially the individuals aged 65 years or older.

The second advantage was the cultural change initiated by the AgICOT project, which dismantled unnecessary barriers that had previously created an artificial separation between hospital and community-based services, as well as between social and healthcare services. This was facilitated by transversal training provided to healthcare professionals, social-health operators, social workers, and administrative staff from public, private, and accredited private social and healthcare organizations not only in the ASL Teramo but across all local health authorities in Abruzzo Region. The training focused on topics related to PHC and TC, totalling to 139.3 credits (nearly the full amount of 150 credits required to fulfill the continuous training obligation for social and health professionals over 3 years in Italy) and involving 815 professionals. This training created favorable conditions for a renewed commitment by professionals to co-design care pathways, based on shared language and mutual understanding of jointly developed protocols and procedures, and the roles of each professional within the processes of continuity of care. This training also facilitated the easier management of professionals, so they can now be deployed across multiple care settings. This is an essential aspect given the upcoming reorganization of social and healthcare facilities in local health authorities, with strong touchpoints such as the creation of unique points of access both to social and healthcare services or the strengthening of the role of multidimensional assessment group, where collaborate social and health professional (19). The third advantage lay in the tools employed. The joint use of the HP and HB in AgICOT definitively linked social and health goals with the effective utilization of all resources provided by all stakeholders involved in the social and healthcare pathway, avoiding duplication of interventions and, consequently, saving time and money. Moreover, the acquisition of a software to ensure interoperability and interconnection between health and social care services' platforms represents a strategic upgrade.

Finally, the fourth advantage was the implementation of case manager profile as companion to assisted individuals and caregivers and as leader of the multiprofessional team. For these reasons, case manager is, maybe, the main key factor of AgICOT's effectiveness.

## 4 Discussion

AgICOT effectively reached goals of international recommendations in terms of continuity of care and integrated care (9, 13), aligning with last WHO recommendations (38).

Data on AgICOT activities have demonstrated the ability of the studied is service to ensure continuity of care to a very large sample by the reorganization of the resource management adopting HP and HB. A key aspect of AgICOT's organizational model was, in fact, its proactive engagement with the target population. Upon receiving a referral to the AgICOT service, the core team promptly reached out to target population, even in cases where these persons might be referred to other services. This offered practical support in identifying the most suitable response to their social or health needs (3). When the referral was deemed appropriate and the individuals chose to participate by accepting the proposed HP and HB services, assisted persons were directly enrolled in AgICOT. From that point, a case manager supported them throughout their care pathway, ensuring personalized care and promoting end-of-life dignity. Consistent with other international experiences (30, 31), the AgICOT case manager served as the primary contact person for both assisted individuals and their caregivers and social and health services, providing essential support to coordinate the provision of an integrated response. The case manager ensured that the social and health care pathway focused on meeting the assisted individuals' needs while addressing any informational or knowledge gaps that hindered the resolution of critical social and health issues. The case manager played a central role in the care process, continuously integrating and coordinating social and health services to fully meet assisted individuals' needs. The case manager also monitored adherence to the shared HP/HB plan over time, adjusting when necessary. The case manager ensured that neither the assisted individual nor the caregiver would face critical moments, such as transitions between different care settings (9, 10), decision-making, or caregiving, alone. It is important to highlight that AgICOT's activity primarily aimed at preserving the self-determination and dignity of assisted individuals and their caregivers, who were often equally frail. This approach aligns with the emphasis of DM 77/2022 (17), which seeks to establish a stronger community-based network for social and health care (18, 19) that is as close as possible to the population, especially vulnerable groups like frail individuals aged 65 years or older. So, the 317 frail individuals aged 65 or over enrolled in the AgICOT project not only benefited of an integrated response provided by social and health care services but have been accompanied daily by the own case manager, single and recognized point of reference within the care pathway.

To achieve these goals, the AgICOT project not only reorganized the continuity of care and transitional care model in ASL Teramo but also reinforced cooperation between hospitals and community-based services; it further strengthened collaboration with reference social services and third-sector organizations. The establishment of this collaborative network, involving all stakeholders from hospital, home care services, and community-based health facilities, positively impacted the economic sustainability of continuity of care. This was achieved through resource sharing and joint planning among all involved social and health actors. This network played a key role in overcoming one of the major challenges currently faced by the Italian social and health system—ensuring access to high-quality care for individuals aged 65 and over (6, 7). This is particularly important given the growing difficulty for this population to navigate social and health services (56, 57), coupled with the reduction in available amid increasing demand for services (1, 2). It is also important to remember how AgICOT service has been implemented iso resources in terms of personnel, structures, and tools, ensuring the sustainability of the service over time.

In AgICOT, the implementation of HP and HB (35, 36) as operational tools among all stakeholders was a crucial strategic move. It facilitated the establishment of a standardized approach that coordinated and harmonized the efforts of diverse actors with differing regulations, characteristics, and procedures. HP and HB enabled stakeholders to jointly define a unified timeline for programming and resource allocation, effectively integrating multiple responses into a single, cohesive solution for assisted individuals. Since 2006, HP and HB have gained traction in the management exclusively of disability conditions and non-self-management in Italy (58, 59), while AgICOT adopted these tools generally for frail individuals aged 65 or over without any restrictions. supporting continuity of care (55, 60, 61). According with this opening,

Due to the 2-year delay in financing the AgICOT project, it overlapped entirely with the structural reform process of the Italian National Health System following the COVID-19 pandemic.

PNRR and Ministerial Decree 77/2022 foresee the establishment of COT (19, 55, 62), structures that perform a function of coordinating the care of the assisted person and connecting services and professionals involved in the various care settings and dialogue with the emergency-urgency network in order to ensure continuity, accessibility and integration between social and healthcare services. COT's organizational model overlaps in a significant way that adopted of the implementation of AgICOT in ASL Teramo, with the exception that AgICOT interacted directly with the assisted individuals and their caregivers and targeted the vulnerable frail individuals aged 65 or over, while the COT is a service for social and healthcare services and serves the entire population. AgICOT can be considered -taking into account the necessary specificities (36, 38)—an early experimental of COT in ASL Teramo. This experimentation allowed for the identification of managerial, organizational, or informative issues for which can be preventively identified possible solutions and applied, if necessary (36, 38), corrective actions,

Moreover, the possibility of use of HP/HB has been extended to the entire population with Italian Ministerial Decree 77/2022. In the progressive wider adoption of these tools to the entire population by hospitals and community-based services, the AgICOT experience could be useful for the development of effective implementation strategies at the national level, and possibly internationally (62). To summarize, AgICOT was designed to improve the synergistic delivery of social and healthcare services, serving as a basis for the application of Mission 6 “Health” in PNRR and DM 77/2022, especially regarding the establishment of COTs.

Furthermore, the number and complexity of cases managed by AgICOT were notably significant, given the high demand in a medium-small size catchment area (287,000 individuals in ASL Teramo, with 24.4% of the population over 65 and vulnerable—an individuals aged 65 years or older dependency ratio of 36.5% in 2021). This achievement represents a substantial quantitative contribution in advancing the ambitious PNRR target of providing home care to 842,000 individuals aged 65 years or older nationwide by the end of 2026, ~4,100 of whom reside within ASL Teramo.

Ultimately, AgICOT fostered progressive professional development of both of social and health professionals, by providing dedicated training, enabling them to actively participate in the definition and implementation of comprehensive social and health care pathways. At the same time, these professionals were able to educate assisted individuals, caregivers, families, and, if necessary, colleagues (63) on communication, coordination, and agreement during transitions. Due to the AgICOT's specific target population, the goal was to ensure that both assisted individuals and their caregivers could actively navigate social and health services until the end-of-life journey with dignity and respected by social and health organizations (56, 64).

## 5 Final considerations

AgICOT achieved two main goals. The first was strengthening the coordination between social and healthcare services in the field of action—ASL Teramo- creatin a strong collaborative network. This network led to the active engagement of economic, professional, and organizational stakeholders (health organizations, social services, no profit organizations, families, caregivers and assisted individuals) in the joint planning and provision of continuity of care for frail individuals aged 65 or over. The second goal, directly linked to the first, was the effective implementation of HP and HB tools by case manager. These tools prevented the duplication of interventions by integrating all service requests into a single social and healthcare response and supporting the case manager, who led the entire multiprofessional team involved in care delivery. This approach promoted the integration of economic, professional, and organizational contributions, ensuring the economic sustainability of the service provision. Indeed, AgICOT has proven to be an effective organizational response for addressing the complex social and healthcare needs of frail individuals aged 65 or over in a sustainable manner. The co-responsibility of different stakeholders (social workers, nurses, general practitioners, caregivers, and so on), who were integral to the AgICOT core team, was the key factor in the AgICOT project's success.

Moreover, AgICOT facilitated the cultural, organizational, and managerial transition toward reformed Italian social and healthcare system, creating favorable generalizable conditions for a shift both from hospital-based to community-based approach and from organization-based to person-based social and healthcare services provision.

Despite all these positive results, it is still necessary to investigate the reasons for the high abandonment rate of AgICOT. It would also be interesting to analyse whether the experience acquired by the operators who worked there are being utilized in territorial health facilities established by DM 77/2022 COTs, as intended. Additionally, the study does not explore the use of HB and HP, nor does it assess the application of competencies and skills acquired by healthcare professionals within the AgICOT setting in COT. Given these gaps, future research could focus on a more in-depth analysis of the COT model, examining its alignment with AgICOT principles, the integration of HB and HP, and the impact of professional skill development on healthcare outcomes. Expanding on these aspects would provide valuable insights and contribute to a more comprehensive understanding and generalization of AgICOT experience.

## Data Availability

The raw data supporting the conclusions of this article will be made available by the authors, without undue reservation.

## Footnotes

^1^AgICOT project was launched in ASL Teramo with resolution no. 431/2022, implementing the resolution of the regional council of the Abruzzo Region no. 919/2021. The working group composed by professionals both of ASL Teramo and University of Florence for the implementation of AgICOT project was first defined with resolution no. 1036/2022, and then with resolution no. 287/2023. The AgICOT organizational model was adopted by order of the Director of the Department of Territorial Activities of ASL Teramo (one of two AgICOT project's scientific referents) no. 0987/2023 (Annex 1).
