# Supplementary material for: Integration of resources in social and healthcare services for ensuring the continuity of care to frail individuals aged 65 or over: an Italian experience
Source: Front Public Health. 2025 May 21;13:1562564. doi: 10.3389/fpubh.2025.1562564 (PMC12135681; doi:10.3389/fpubh.2025.1562564)
Supplement: Supplementary file 1 [file Data_Sheet_1.docx]

**Annex 1 – Responsibility matrix of AgICOT**

| ***Phases*** | ***Description*** | ***General practitioner*** | ***Transitional care service*** | ***Social Services*** | ***Home care service*** | ***Administrative*** | ***AgICOT Responsible*** | ***AgICOT Project Management*** | ***Team Core*** | ***Case Manager*** | ***Specialists*** | ***Assisted individual*** | ***Caregiver*** | ***Legal representative*** |
| --- | --- | --- | --- | --- | --- | --- | --- | --- | --- | --- | --- | --- | --- | --- |
| ***Service***  ***activation*** | The request is made by request on the appropriate form | R | R | R | R |  | I | I |  |  |  |  |  |  |
| ***Request***  ***acceptance*** | Request reception |  |  |  |  | R | I | I | C |  |  |  |  |  |
|  | Control on personal data completeness |  |  |  |  | R | I | I | C |  |  |  |  |  |
|  | Entry of assisted person in informative platform (personal data, GP, caregiver and/or legal administrator). |  |  |  |  | R | I | I | C |  |  |  |  |  |
|  | Insertion of the case in the Team Core work calendar in the first available slot. |  |  |  |  | R | I | I | C |  |  |  |  |  |
|  | Registration of begun clinical evaluation process informative platform |  |  |  |  | R | I | I | C |  |  |  |  |  |
| ***Request***  ***evaluation*** | Clinical Evaluation of received documentation. |  |  |  |  |  | I | I | R |  |  |  |  |  |
| ***Request***  ***appropriateness***  ***evaluation*** | Because of age and the received social and health documentation, the request appropriateness is assessed.  If the request is for a patient over 65 years of age with a Brass Index >= 10, it is considered appropriate. If the patient is under 65 years of age or Brass Index < 10, request is not appropriate, and the reporting service is contacted. |  |  |  |  |  | I | I | R |  |  |  |  |  |
|  | If the advice of specialists is necessary but not available at the time, or the received documentation is not sufficient to set up HP/HB, the necessary consultations will be activated, the missing documentation will be found and a new meeting will be scheduled. |  |  |  |  |  | I | I | C | C | R |  |  |  |
| ***Health Plan and***  ***Health Budget***  ***definition*** | If it is possible to proceed, the Team core drafts HP/HB |  |  |  |  | I | I | I | R | C |  |  |  |  |
| ***Case manager***  ***identification*** | Based on prevailing needs of the assisted person, the case manager for taking charge is identified. |  |  |  |  | I | I | I | R | C |  |  |  |  |
| ***Communication of***  ***taking charge*** | Notice of the GP, the social worker, and at least one between assisted person, caregiver and legal administrator of the taking charge by AgICOT, explaining the next steps (monitoring and follow-up). | I |  | I |  | I | I | I | C | R |  | I | I | I |
| ***Monitoring*** | Monitoring of assisted person conditions according to the methods and timing established by the HP/HB |  |  |  |  | I | I | I | C | R |  |  |  |  |
| ***Care*** | Booking social and health services provided in the HP by discussing with the patient and/or caregiver and/or legal administrator and giving them notice (by phone, text message and e-mail) of the scheduled date for the scheduled services. |  |  |  |  | I | I | I | I | R |  | C | C | C |
| ***Revaluation*** | If during monitoring or following a report by the patient/caregiver/GP/social worker, the need to change the HP/HB is found, the case manager assesses if substantial changes to the HP/HB are necessary or not |  |  |  |  | I | I | I | C | R |  |  |  |  |
|  | If substantial changes are necessary, a new core team meeting will be convened to proceed with the change of HP. | I |  | I |  | I | I | I | R | C |  | I | I | I |
|  | If substantial changes are not necessary, case manager provides for additions by reporting the changes in the monitoring diary and informing GP/Social worker and at least one patient, caregiver and legal administrator. | I |  | I |  | I | I | I | C | R |  | I | I | I |
| ***Follow-up*** | Reporting of monitoring results and supervening additions/changes to the HP/HB |  |  |  |  | I | I | I | I | R |  |  |  |  |

Legend: *R= Responsible* *C=Consultant*  *I=Informed*

**AgICOT responsible** assesses the progress of the activities provided by the AgICOT proposes any improvement actions in agreement with the AgICOT Project Management through its functional articulations

**AgICOT Project Management** is responsible for creating/managing/monitoring/reporting the AgICOT organizational model. It consists of ***project manager in medical area*** and of ***project manager in health and social professionals***

**Team Core**:

- ***Physicians*** who coordinate the AgICOT team core, assess the appropriateness of the request in a multi-professional team, support the integration of AgICOT with hospital and territorial health services, define, optimize, activate and monitor the most appropriate HP among the different care paths.
- ***Social Workers*** is responsible for the connection activities between the National Health Service and the local social services for the configuration of the network of services in favor of the assisted person and advocacy activities in the civil/legal paths.
- ***Coordinator of health professionals*** is responsible for the continuity of care between different settings.

**Case manager** is identified between Physicians, Health Professions and Social worker based on prevailing needs of the assisted person. Case Manager becomes the assisted person/caregiver’s contact person, supervises and coordinates HP/HB reporting any changes to Team Core.

**Administrative** is responsible for the control of administrative bureaucratic activities such as the necessary and essential data for the drafting of the HP/HB

**Annex 2 – Flowchart AgICOT**

AgICOT’s workflow diagram has been extracted from the procedure developed by ASL Teramo.

**Annex 3 - Scheme of semi-structured interview**

| **AREAS** | **QUESTIONS** |
| --- | --- |
| **Strategic purposes on continuity of care in the field of action** | - What are the company policies adopted to ensure continuity of care in general? - What were the objectives, with respect to the needs of the territory, of these policies? - Are there any company policies adopted to ensure continuity of care, in particular for frail individuals aged 65 or over? If so, which ones? - What are the objectives, with respect to the needs of the territory, of these policies? - What are the services involved? |
| **AgICOT establishment** | - What is the organizational model developed for AgICOT? - What management tools have been made available to the service? - What technological tools have been made available to the service? - Which professionals have been made available to the service? - Has there been specific training of the staff? |
| **AgICOT activity** | - How are reports handled? - How are communications to the beneficiaries of the service or assisted individuals/caregiver(s)/ other figures managed? - How are communications with colleagues outside the Teramo ASL managed? - How are information flows managed? |
| **AgICOT impact** | - What has been the impact of AgICOT in terms of integrating social and health care? - What has been the impact of AgICOT in terms of strengthening the continuity of care? |
